# Multipoint identity-by-descent computations for single-point polymorphism and microsatellite maps

**DOI:** 10.1186/1471-2156-6-S1-S34

**Published:** 2005-12-30

**Authors:** Anthony L Hinrichs, Sarah Bertelsen, Laura J Bierut, Gerald Dunn, Carol H Jin, John S Kauwe, Brian K Suarez

**Affiliations:** 1 Department of Psychiatry, Washington University School of Medicine, St. Louis, Missouri, USA; 2 Department of Genetics, Washington University School of Medicine, St. Louis, Missouri, USA; 3 Division of Biology and Biomedical Sciences, Washington University School of Medicine, St. Louis, Missouri, USA

## Abstract

We used the LOKI software to generate multipoint identity-by-descent matrices for a microsatellite map (with 31 markers) and two single-nucleotide polymorphism (SNP) maps to examine information content across chromosome 7 in the Collaborative Study on the Genetics of Alcoholism dataset. Despite the lower information provided by a single SNP, SNP maps overall had higher and more uniform information content across the chromosome. The Affymetrix map (578 SNPs) and the Illumina map (271 SNPs) provided almost identical information. However, increased information has a computational cost: SNP maps require 100 times as many iterations as microsatellites to produce stable estimates.

## Background

Traditionally, the mainstay of linkage has been use of highly polymorphic microsatellite markers. The ultimate goal would be completely polymorphic markers – each parent would have two uniquely occurring alleles. A highly polymorphic microsatellite provides a great deal of segregation information at a particular locus. At the other extreme, single-nucleotide polymorphisms (SNPs) usually have only two alleles (more alleles are possible but uncommon) and alone provide much less information for segregation. Because SNP typing is less expensive, and available at a finer density than microsatellites, the use of dense SNPs in the place of microsatellites for linkage analysis is being investigated using data from the Collaborative Study of the Genetics of Alcoholism (COGA). Because segregation is at the heart of any linkage analysis, we examined IBD (identity by descent) matrices to compare the information content of SNPs versus microsatellites for linkage. We used the LOKI software [1,2] to create the matrices after a set of preliminary tests to determine the appropriate number of iterations. However, due to time and computational constraints, we have restricted our attention to chromosome 7. Although the matrices are created irrespective of phenotype, published results from the COGA project have shown linkage with multiple phenotypes on this chromosome [3]. Other members of our group used the matrices to replicate some of these findings [4].

## Methods

### Sample

Approximately 1,300 individuals previously typed with a microsatellite screen were typed with 4,720 SNPs from the Illumina linkage panel and 11,120 SNPs from the Affymetrix mapping array. These individuals were from 143 pedigrees with an average of 9.5 individuals typed per pedigree (range: 5–27). The sample was 77% White, 13% African American, and 10% from other ethnicities.

### Analyses

In the presence of non-genotyped founders, allele frequency estimates are of paramount importance for IBD estimation. The large differences in allele frequencies between Whites and African Americans for microsatellites have been well established. Our group identified similar differences for allele frequencies of SNPs [5]. Because of this all of our analyses were restricted to 112 of the 143 pedigrees in which the entire pedigree was unambiguously White (as determined by self report and STRUCTURE) [5]. These pedigrees had an average of 9.3 individuals typed per pedigree (range: 5–27). Maximum likelihood estimators (MLE) for allele frequencies for both SNPs and microsatellites were computed using the 'freq mle' option in SOLAR [6].

The generation of IBD matrices with microsatellite markers has been addressed through a number of different techniques. We are using multipoint IBD (mIDB) as our standard because of the low information provided by a single SNP. Although a new version of GENEHUNTER [7] has recently been released to deal with SNP markers, the large size of some of these pedigrees required extensive trimming because the basic algorithm still computes all possible inheritance vectors. We therefore decided to use the LOKI software to generate IBD matrices.

**Figure 1:**
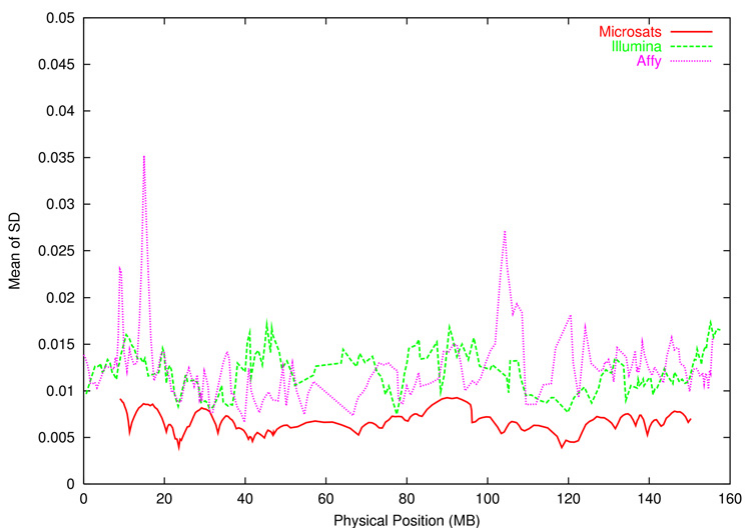
Mean of standard deviation of 2φ (10,000 iterations).

**Figure 2:**
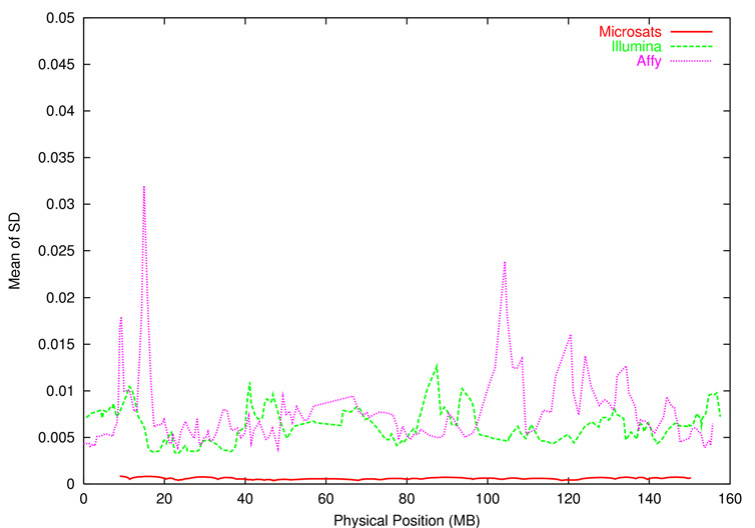
Mean of standard deviation of 2φ (1,000,000 iterations).

LOKI uses Markov chain Monte Carlo (MCMC) methodology to repeatedly sample possible segregation patterns. However, determining appropriate run length (number of iterations) is important for the accuracy of the IBD estimates. Using three separate sets of markers: (microsatellites, Affymetrix, and Illumina) we compared the average standard deviation of "phi2" (twice the kinship coefficient) between each pair of individuals in each pedigree at each centimorgan position on chromosome 7 for 10 replicates (from 10 different starting seeds) with 10,000, 100,000 and 1,000,000 iterations per replicate. To compare this information on different maps, we translated the genetic positions of the markers to the physical position based on NCBI build 34.3 (Figures 1 and 2). Ultimately, we used 1,000,000 iterations to compute IBD estimates for each White pedigree for the SNP maps. These computations were performed on a Beowulf-class computer cluster consisting of 60 dual processor nodes (25 dual Pentium II 350 MHz, 8 dual Pentium II 550 MHz, 18 dual Pentium III 800 MHz, 9 dual Pentium III 1,000 MHz), each with 512 MB of RAM; this provides an effective 18 GFLOP/s capacity (based on the Linpack benchmark [8]).

Using the resulting matrices, information was first computed on sibships (regardless of phenotype) using the method presented in Kruglyak and Lander [9]: for *N *sibling pairs (*i*, *j*) at position *x*, we compute



**Figure 3:**
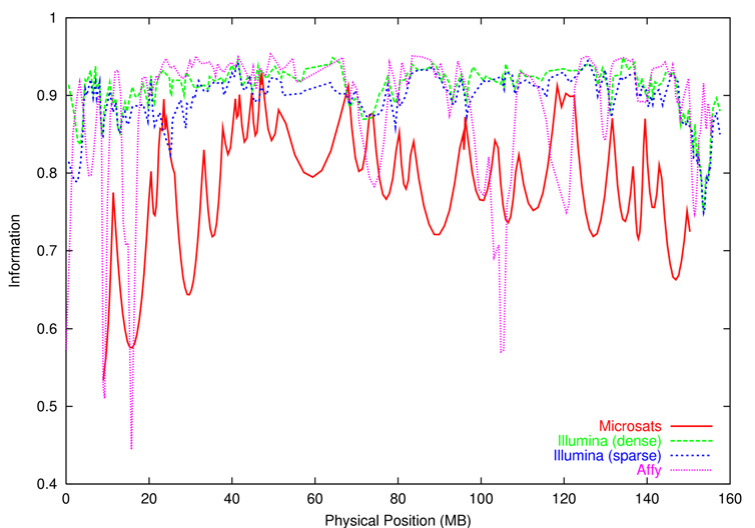
Information for sibling pairs.

At each chromosome position for each sibling pair, the variance in IBD 0, IBD 1, and IBD 2 estimates is divided by the variance in the absence of marker information (for siblings, this is 0.5). The mean of this measure is subtracted from 1. If the posterior IBD status is known with certainty for all pairs, the variance is 0 and the information is 1. This measure was then computed on all relative pairs except for parent-offspring, where the prior variance is 0 (since Pr(IBD = 1) = 1.0), notwithstanding a new mutation. The results for the sibling pairs are presented in Figure 3.

Finally, we note that LOKI assumes the markers are in linkage equilibrium (LD). However, substantial pair-wise LD exists between the SNPs, especially in the Affymetrix map. Considering all pairs of Affymetrix SNPs on chromosome 7, 59 pairs have a correlation greater than 0.9. For Illumina, 13 pairs have a correlation greater than 0.9. Recent work [10] suggests that when parental genotypes are unavailable, this linkage disequilibrium between SNPs can artificially inflate IBD estimates and may also inflate estimates of information content because the inflated IBD estimate has an artificially high precision. Although many of the COGA pedigrees have parental genotypes, we conjectured that a less dense SNP map might still contain most of the information. The Illumina map began with lower LD so we chose to reduce it (rather than the Affymetrix map) because fewer deletions would be required. We constructed a subset of 166 markers from the Illumina dataset on chromosome 7. Markers were deleted from the dataset if they had a D' value greater than 0.1 with nearby markers. When possible, we retained markers with the highest possible minor allele frequency. The results are presented in Figure 3.

## Results

Due to the large size of some of these pedigrees, software which computes all possible inheritance vectors (such as GENEHUNTER or Merlin [11]) has excessive memory requirements unless the pedigrees are pruned. Because all genotyped individuals can be used for quantitative trait analysis, this was deemed unacceptable.

In general, MCMC software trades these memory requirements for substantially greater CPU usage. We thus chose LOKI for IBD matrix generation, but this software requires a choice of run length (number of iterations). The initial tests to determine an appropriate number of iterations for the generation of IBD matrices with LOKI shows that the higher density of the SNP maps greatly slows the MCMC process. In particular, while 10,000 iterations for microsatellites shows only slightly higher average variance of phi2 than one 100,000 iterations, the variance for SNPs is still quite high with 100,000 iterations. We ultimately used 1,000,000 iterations for the SNP datasets. Figures 1 and 2 show that 1,000,000 iterations for the SNP map produces about the same variance of phi2 as 10,000 iterations of the microsatellite map. However, there are still peaks of high variance, especially in the Affymetrix map.

The information content results for microsatellites show substantial dips between markers. Both the Affymetrix and Illumina map provide a higher and much more uniform level of information. There are several sharp dips in the Affymetrix map, but this may be due to the IBD generation failing to converge; the locations of reduced information correspond to the locations of higher variance in Figures 1 and 2. Although the Affymetrix map has more than twice as many SNPs as the Illumina map, information is not significantly higher (*p *= 0.3). We also note that the "sparse" Illumina map (with an average intermarker spacing of 1.1 cM) contains substantially more information for sibships than the microsatellites and nearly as much information as the full Illumina map (with an average intermarker spacing of 0.69 cM). The information for relative pairs is uniformly lower than sibling pairs for all four map sets (results not shown). This may be due to the greater number of missing founders when considering the extended pedigrees as opposed to sibships.

## Discussion and Conclusions

Our results show that the information provided by dense SNP maps is generally higher and more uniformly distributed than with standard microsatellite panels composed of about 400 markers. This increased information comes at a cost of increased computational complexity. At least 100 times as many iterations are required and each iteration took 10–20 times longer for the SNP maps as for the microsatellite. For example, 100,000 iterations took 3.4 hours for the microsatellites, 30 hours for the Illumina SNPs, and 68 hours for the Affymetrix SNPs. While the increased time for each iteration is likely due to the increased number of markers, the increase in required iterations may be due to the reduced information of the SNP markers. This could be tested by comparing convergence with a dense microsatellite map.

The Affymetrix map contains regions of reduced information, corresponding to the same locations where variance of phi2 is high. We examined the Affymetrix SNPs around the largest peak (at 15 Mb) and found that they were not significantly different from other Affymetrix SNPs on chromosome 7 in terms of density, heterozygosity, LD, or missing data rate. Other possible explanations include an increase in Mendelian-compatible genotyping errors or incorrect maps (either spacing or marker order). These possibilities could be tested in additional datasets to see if convergence problems existed at the same location.

Although the Affymetrix map consists of more than twice as many SNPs as the Illumina map, increased density of SNPs in the Affymetrix map does not appear to provide more information. However, many of the SNPs in the Affymetrix map have fairly low heterozygosity [5]. We also observed that a subset of the Illumina map provided nearly as much information as the full map. Although the best solution for markers in LD is probably to modify existing software to haplotypic information, it appears that simply removing SNPs may be a useful interim procedure.

These data suggest that SNPs are a cost effective and informative replacement for microsatellites for linkage analysis. Although the computational burden is substantially greater for IBD computations, the resulting information is higher and more uniform. Although estimates of IBD and information content may be elevated when markers are in linkage disequilibrium and parents are untyped, further tests also suggest that a less dense map would provide nearly the same level of information.

## Abbreviations

COGA: Collaborative Study on the Genetics of Alcoholism

IBD: Identity by descent

LD: Linkage disequilibrium

MCMC: Markov chain Monte Carlo

MLE: Maximum likelihood estimators

SNP: Single-nucleotide polymorphism

## Authors' contributions

ALH formatted the data files, analyzed the data, and drafted the manuscript. JSKK provided analyses identifying White subgroups. SB, LJB, GD, CHJ, and BKS participated in the design and coordination of the study. All authors read and approved the final manuscript.
